# Mind the Gap: Wearable Lactate and Glucose Monitors for Hospitalized Patients

**DOI:** 10.7759/cureus.78536

**Published:** 2025-02-05

**Authors:** John Guzzi, Florian Falter, Avinash B Kumar, Albert C Perrino, Jr

**Affiliations:** 1 Anesthesiology and Critical Care Medicine, Yale School of Medicine, New Haven, USA; 2 Anesthesiology and Critical Care, Royal Papworth Hospital, Cambridge, GBR; 3 Anesthesiology and Critical Care Medicine, Vanderbilt University Medical Center, Nashville, USA; 4 Anesthesiology, Yale School of Medicine, New Haven, USA; 5 Anesthesiology, VA Connecticut Healthcare System, West Haven, USA

**Keywords:** early warning scores, exercise physiology, glucose metabolism, glycolysis, hyperlactatemia, inpatient medicine, lactate metabolism, lactic acidosis, monitors, wearable technology

## Abstract

Continuous and non-invasive monitoring of metabolic parameters, particularly glucose and lactate, represents a critical advance in inpatient medicine where current technologies fall short. Wearable sensors are widely available for glucose, and are commonly and successfully used in ambulatory settings. More recently, wearable sensors have become available for lactate, and these devices have a proven role in exercise physiology literature. In the inpatient setting, metabolic monitoring of glucose and lactate is primarily done via invasive blood draws, which is resource-intensive and leads to delays in data collection. This new frontier of wearable continuous monitors has the potential to transform inpatient care by rapidly detecting metabolic changes and eliminating existing gaps in monitoring. Further, these advances in wearable technology open pathways for integrating lactate and glucose metrics into Early Warning Scores (EWS), enhancing their predictive capabilities. The adoption of wearable lactate and glucose in the inpatient setting has the potential to revolutionize a clinician’s ability to identify vulnerable patients at risk for deterioration while guiding precision treatment.

## Introduction and background

The armamentarium available for monitoring various physiologic parameters has taken great strides. In contemporary clinical practice, continuous, noninvasive assessment of critical parameters including arterial blood oxygen saturation and pressures, electrocardiography, fluid responsiveness, and respiration are commonly employed in at-risk patients. Monitoring technology of key metabolic parameters, however, has lagged in the clinical setting. They are too often invasive, difficult to acquire, and available only on a periodic basis. This monitoring discordance leaves the clinician compromised, where early warning of deterioration is missed, the diagnosis inaccurate, and treatment decisions misdirected. 

In this article, we describe how the advent of wearable metabolic monitors, specifically for lactate and glucose, provides new opportunities to improve care and outcomes in these at-risk patients. Further, these devices become invaluable when viewed in the context of recent research, much of it gleaned from exercise physiologists, which have informed our understanding of the body’s adaptive response to stress. This research upends traditional concepts of lactate as a metabolic waste product resulting from hypoxic tissue. Rather, it demonstrates the key role lactate plays, along with glucose, in fueling the body’s energy needs during stress, whether that is due to intense exercise or pathological illnesses. As such, wearable metabolic technology, much as it has for athletic training, becomes imperative in the clinical setting to optimize treatment and outcomes.

## Review

Glucose and lactate cycling: two ends of one metabolic system

Glucose and lactate are intricately linked in the body’s energy system in a cycle fundamental to cellular metabolism. Glucose, stored as glycogen, is a primary energy substrate that is metabolized in the cytosol by glycolysis to produce pyruvate and energy in the form of adenosine triphosphate (ATP). Under normal oxygen conditions (aerobic metabolism), pyruvate is further oxidized in the mitochondria via the citric acid cycle and electron transport chain, producing ATP efficiently. However, when oxygen availability is limited, such as during hypoxia or in conditions of metabolic stress, pyruvate accumulates and is converted into lactate through anaerobic glycolysis.

Lactate is produced in all cells with dominant production from skeletal muscles (25%), skin (25%), brain (20%), intestines (10%), and erythrocytes (20%). The lactate released into the bloodstream is taken up and metabolized almost entirely by the liver (60%) and kidneys (30%) back to glucose via gluconeogenesis [1]. Thus, lactate serves as both an essential metabolite and a substrate for energy storage. 

During periods of stress, such as intense exercise or critical illness, this interplay between glucose and lactate becomes even more important. According to traditional teaching, elevated lactate levels in these conditions were considered the result of tissue hypoxia. This interpretation, known as type A lactic acidosis, positions lactate as metabolic waste created in scenarios of hypoperfusion and/or impaired microcirculation, such as shock. Shock ultimately represents a microcirculatory failure that drives anerobic metabolism accelerating the production of lactic acid [2]. Hyperlactatemia is a common feature of shock and is correlated with the severity of shock and patient mortality [3-11]. Accordingly, lactate levels are widely utilized to stratify a patient’s risk, guide treatment, and assess outcomes.

In contrast with type A lactic acidosis, type B lactic acidosis describes an elevation in lactate without hypoxia or hypoperfusion. Type B lactic acidosis is typically attributed to mitochondrial dysfunction, drug or toxin exposure, liver disease, or conditions that accelerate the rate of glycolysis (e.g, severe infection, sepsis, shock, and administration of exogenous beta-adrenergic stimulants) [12, 13]. The significance of type B lactic acidosis is often overlooked, and the bulk of clinicians’ attention is paid toward the concern of type A (hypoxic) pathology.

However, emerging data from contemporary physiology challenges this dichotomy between hypoxic and non-hypoxic lactate accumulation and suggests that the truth is not so simple [14]. For example, the hyperlactatemia seen under intense exercise occurs despite adequate oxygen levels in the muscle cells. Rather than a marker of hypoxia, lactate elevation represents an effective means of meeting the body’s increased energy demands over and above that provided by mitochondrial oxidation. In essence, hyperlactatemia in these conditions represents the deployment of an alternative fuel source and a healthy adaptive response.

The vital role that lactate plays as a key intermediary for cell signaling, energy utilization, and adaptation in whole body energetics is more comprehensively captured by the lactate shuttle hypothesis. In this model, lactate is shuttled between cells and organs as a signaling molecule and fuel source. The lactate shuttle hypothesis describes a give-and-take relationship where “driver cells” (such as exerted myocytes) produce lactate, which is utilized in “recipient cells.” In this model, locally-produced lactate is not only a key energy derivative of anaerobic metabolism. It is also a long-distance source of fuel: peripheral lactate can be utilized as a fuel by central organs such as the liver, heart, and brain [15-17]. This dynamic is as true in healthy athletes undergoing physiologic testing as it is in critically-ill patients [18-20].

The lactate shuttle hypothesis upends the conventional, often automatic and unconscious, teaching in clinical medicine that elevated lactate marks hypoperfusion. The production of lactate occurs under both anaerobic and aerobic conditions in a dynamic fashion that is not simply a binary process classified as “type A” or “type B.” Its presence is important for energy production and recycling. In effect, the glucose/lactate cycle is the fulcrum of metabolic integration.

This biochemical framework recognizes that lactate and glucose are both essential and interchangeable forms of energy currency within the body. Alterations in glucose and/or lactate are often the first evidence of metabolic adaptation to an acute stressor, and recognizing this fact allows clinicians to interpret changes in health status with greater sensitivity and nuance. Further, rather than interpreting a lactate level as being “positive” or a glucose level as being “elevated,” attention is owed to the rate of rise and the rate of clearance, recognizing that these trends are often more important than an absolute number. The primacy of lactate trends has important implications not only for early detection and informing diagnosis but also for guiding initiation and cessation of therapies, many of which can cause harm when misapplied (e.g., excessive fluids, vasopressor therapy, etc). 

This framework also emphasizes the give-and-take relationship that exists between glucose and lactate. For example, hyperlactatemia can be directly caused by hyperglycemia, and vice-versa [21]. This approach opens the door to tailored therapeutic strategies aimed at an underlying physiologic context that considers a patient’s global metabolic state.

In clinical practice, the metabolic state of a patient is often inferred from isolated, or at best, episodic lactate and glucose measurements. Conventional episodic monitoring is labor-intensive (requiring a fresh blood sample and laboratory resources) and is often done in intervals measured in hours. The timing of these laboratory tests creates gaps where clinical changes go unrecognized and treatments are either delayed leaving clinicians trying to catch up to a deteriorating condition or inappropriately continued past their benefit and to the point of harm. Wearable, continuous monitors of glucose and lactate have the potential to revolutionize inpatient care for both critically and non-critically ill patients by the elimination of these gaps.

The advent of continuous glucose monitors, as discussed below, has advanced the clinical care of many patients in the ambulatory setting, especially those affected by type one diabetes. Combining them with continuous interstitial fluid-based lactate monitors, which are under development at the time of this writing, creates the potential to dramatically improve the sensitivity for the detection and management of unwell or threatened hospital patients.

Fundamentals of wearable sensor technology

Sensor technology is constantly advancing. Only a few years ago, wearable technology might have attracted the attention of physiologists and some clinicians, but its use was eyed with great suspicion due to the lack of rigorous validation. However, the commercial and clinical success of wearable monitors, particularly flash glucose monitoring sensors, has proved a game changer. The adoption of this technology has opened the door to expanding the role of metabolic monitoring technology into other fields of medicine.

The use of non-invasive technologies for analyzing biological fluids, which includes both wearable glucose and lactate monitors, has been investigated extensively. Tears, saliva, sweat, and interstitial fluid (ISF) can all be analyzed for various metabolites and compounds in a non-invasive way.

Biomarkers measured in saliva show a good correlation with blood levels. Continuous sampling is possible by fitting mouthguards with sensors. Saliva, however, is prone to contamination with food intake or oral hygiene [22]. This technology has not been validated in hospitalized patients and does not seem entirely practical or comfortable for continuous use.

Amperometric sensors incorporated into contact lenses can reliably and reproducibly detect glucose even at low concentrations. Even if this were feasible for other biomarkers than glucose, the difficulties associated with extracting tears for continuous monitoring currently make this an impractical proposition [23].

**Figure 1:**
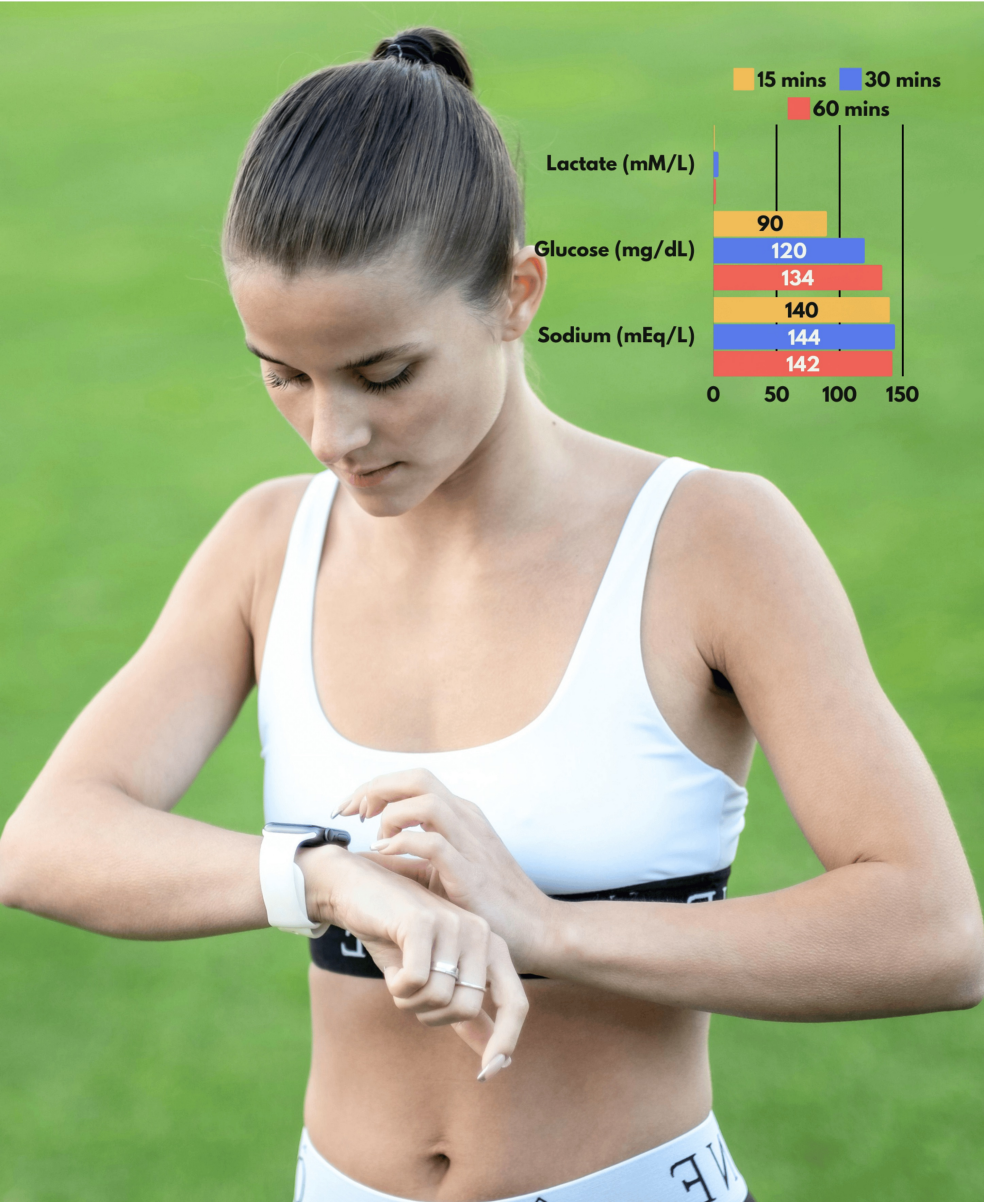
An illustrative example of a wearable device that integrates with an athlete’s watch to monitor lactate, glucose, and sodium on an every-fifteen-minute basis. This figure is the original work of the authors. Photograph by M. Shevchenko from Pexels with Creative Commons Zero (CC0) license.

Sweat is rich in biological information and is easy to harness with wearable sensor technology [24]. There is a suggestion that the glucose concentration in sweat may correlate with that in blood, potentially making it useful for blood glucose monitoring [25]. Lactate concentrations in sweat and blood are closely correlated [26]. The fact that sweat secretion rate and lactate concentration are independent of each other, makes it an attractive target for non-invasive monitoring during exercise and critical illness [27]. Figure 1 demonstrates the utility of a sweat-based monitor in an athletic setting. However, it is important to note that the relationship between sweat lactate values and blood lactate values can be discordant, especially under exercise conditions where there is the most experience utilizing wearable lactate monitors [28]. While sweat-based monitoring has limitations in the inpatient arena, the technology is an excellent proof of concept.

Interstitial fluid (ISF) - compared to other peripheral biofluids such as saliva, sweat, and tears - is a particularly rich source of soluble bio-analytes, including proteins, peptides, metabolites, and nucleic acids, which exhibits a close correlation with blood. As such, ISF holds promise as a blood proxy. The most common molecules that are amenable to these technologies include glucose, lactate, ketones (beta-hydroxybutyrate), ethanol, electrolytes, and even certain medications such as vancomycin and levodopa [29].

ISF is easily sampled via integrated wearable microneedle arrays that are each about one-fifth the width of a human hair. Wearing the device is not painful - the microneedles barely penetrate the skin surface to sense biomolecules in interstitial fluid.

These devices have enzymatic electrodes and are based on the action of enzymes that catalyze redox reactions by creating an electric current with measurable voltage, which in the case of glucose monitors are dependent on glucose concentrations [30]. A very similar technology can be applied to derive a near-continuous lactate concentration from the interstitial fluid. The data is then transmitted to a wearable device and/or mobile phone so one can track changes to the glucose level or lactate level in real-time. This data can also be integrated into the electronic medical record (EMR).

A summary of key technologies underlying wearable monitoring devices is presented in Table 1.

**Table 1:** Comparison of wearable microarray sensors Wearable microarray sensors most commonly work either via enzymatic sensors or ion-sensitive electrodes. These two mechanisms of action have limitations that must be considered when targeting specific molecules such as glucose or lactate [31].

	Ion-Sensitive electrodes	Enzymatic sensors
Mechanism	Senses changes to the Nernst potential in the electrochemical cell	Detects electrical current change generated by the products of enzymatic reaction
What can be measured	Electrolytes (sodium, potassium, magnesium, calcium)	Small molecules that can be enzyme substrates: glucose, lactate, creatinine, urea
Technology limitations and confounders	Technology works mostly with charged particles (ions)	Acetaminophen and uric acid can potentially interfere with the sensors

Fundamentals of Lactate Sensors

The concept of reliably and continuously measuring lactate using wearable sensors is very well established in training elite athletes. Although there are several other physiological parameters such as heart rate, VO2 max, or ventilatory threshold to categorize the body’s response to exercise intensity, lactate is considered the most sensitive parameter [32]. The introduction of the lactate threshold, the point where lactate accumulation increases sharply [33], to optimize training regimens was made possible through the emergence of wearable devices. 

The currently available wearable lactate biosensors are designed for the professional athlete performance market and make use of sweat analysis. They are often integrated into wrist-worn devices such as smartwatches, but increasingly they are being integrated into garments [34]. It is important to note that sweat concentrations vary between different areas of the body [35]. A device using ISF to sample the analyte would be able to eliminate this limitation. 

Several endeavors are underway to create wearable ISF lactate sensors based on already available technology used for glucose monitoring [36]. The development of ISF-based wearable lactate monitors represents a frontier in device manufacturing with potentially enormous clinical applicability for hospitalized patients.

Fundamentals of Glucose Sensors

Although sweat, tear, or saliva analyses are feasible, ISF has emerged as the prime target for BG monitoring. Due to the advantages of continuous monitoring, of the seven million patients with type 1 diabetes in the USA, about 35-50% wear a flash glucose monitoring system [37]. 

The good correlation of glucose levels between ISF and blood has been confirmed in several studies [38]. Microneedle devices have successfully been commercialized in flash glucose monitoring systems.

These systems make use of inserting a 0.4 mm disc-mounted fiber about 5 mm into the back of the upper arm. The fiber draws ISF to the sensor, which analyses the glucose levels automatically at regular intervals. The lag between BG and glucose measured in the ISF is about 10 minutes [39]. The disc, which secures the fiber to the skin, can house means of communication with a reader or a hospital laboratory information system middleware to allow integration into an EMR.

Applications for hospitalized patients

Continuous Glucose Monitoring

Continuous monitoring of both glucose and lactate levels has the potential to provide new insight into a patient's metabolic state, opening the door to earlier and more targeted interventions. Glucose in the monitoring in the ICU is a critical component of patient management for all patients, but especially those with risk factors for glucose dysregulation such as individuals with diabetes, sepsis, recent operations, or acute illness. Hyperglycemia and hypoglycemia are both associated with adverse outcomes in critically ill patients. In addition, insulin therapy carries significant and life-threatening risks, making accurate and timely glucose monitoring essential for guiding treatment decisions [40, 41].

Wearable glucose monitoring has the potential to revolutionize early detection of clinical status changes in at-risk hospitalized patients by providing real-time, dynamic insights into metabolic fluctuations. Traditional methods, such as intermittent blood glucose testing, are often invasive, labor-intensive, and may not provide real-time data to effectively manage rapid fluctuations in glucose levels risking undetected, critical changes in glucose levels between measurements.

In contrast, CGM allows for continuous tracking of glucose levels, offering immediate feedback on both hyperglycemia and hypoglycemia, which are often early indicators of metabolic distress. These wearable glucose sensors have been widely adopted for ambulatory patients and their use for hospitalized patients is compelling. Hospitalized patients have many obvious factors that can negatively impact their glucose homeostasis: parenteral nutrition, steroid administration, post-operative status, infections, sepsis, medication administration (such as exogenous beta-adrenergic agonists), and more. Tight control of glucose levels in these patients has been established as a standard of care. There is a wealth of evidence supporting the benefit of continuous glucose monitoring in the ambulatory setting: improved hemoglobin A1C values, decreased hypoglycemic events, improved adherence to treatment plans, and improvement in patient-centered outcomes [42-50]. With this in mind, the potential value of extending this use to inpatient medicine becomes evident.

Hyperglycemic and hypoglycemic events are harmful for hospitalized patients [40], and a continuous glucose monitor aids in the early detection of metabolic derangement. Critically ill patients often have rapidly fluctuating glucose levels due to stress, infection, medication, or post-operative status, and stand to benefit from vigilant use of a continuous monitor. The use of a continuous glucose monitor also has promise in the management of non-critically ill patients, as it may aid in the detection of vulnerable patients at risk of deterioration in an out-of-ICU setting. Abnormal glucose patterns can serve as early warning signs for conditions like sepsis, shock, or insulin resistance, prompting quicker intervention [51].

Moreover, CGM systems can integrate with hospital monitoring technologies, triggering alerts when glucose levels reach dangerous thresholds. This can improve patient outcomes by reducing complications associated with delayed recognition of glucose imbalances. Ultimately, the ability of CGM to provide continuous, actionable data represents a significant advancement in personalized and proactive care for hospitalized patients.

Continuous Lactate Monitoring

Hyperlactatemia is a hallmark characteristic of shock states and the degree of increase in lactate concentrations is directly related to the severity of the shock state and to mortality rates. This role of lactate as a predictor has been demonstrated in patients with septic shock [3-6], cardiogenic shock [11], patients in the ICU [7,8], patients with pulmonary embolism [9], and patients who were admitted following a high-risk surgery [10]. 

A landmark early-goal-directed therapy study highlighted the endpoints of resuscitation as normalization of mixed venous oxygen saturation, arterial lactate concentration, base deficit, and pH in the management of septic shock [52]. These principles have been extrapolated for hemodynamic resuscitation beyond the emergency rooms and ICUs. Ongoing measurement of blood lactate levels (every 2 to 4 hours until normalization) is useful to identify ongoing impairment in tissue perfusion not just in the intensive care or emergency room settings [53, 54]. Although the bulk of published studies has largely been centered on septic patients, several other studies have reported on the usefulness of serial blood lactate levels in other cohorts of acutely ill patients such as congestive heart failure, trauma, and post-surgical patients to name just a few.

Serial lactate measurements serve as a guide to the bedside clinician that the macrocirculatory resuscitation is progressing in the anticipated direction. Not every elevated lactate level requires an additional fluid prescription, and not every normal lactate level means a patient is well [55]. The time trends in lactate levels (even when decreasing toward a normal level) provide useful information on lactate clearance rates and a patient’s response to the prescribed therapy.

Lactate levels have a strong prognostic value and are very useful at the start of resuscitation and the assessment of serial lactate levels is useful as a linear indicator of the progress of resuscitation [56]. Serial lactate measurements are better prognosticators than a single lactate measurement in the shock state [57]. Lee et al showed that both 6-hour lactate levels and 6-hour lactate clearance had a positive prognostic value for predicting the 30-day mortality in patients with sepsis and septic shock in accordance with the Sepsis-3 definitions [58].

Vincent et al published a systematic review of the value of blood lactate kinetics in critically ill patients, which included data from 96 studies spanning patient cohorts of post-cardiac surgery, sepsis, post-cardiac arrest, and cardiogenic shock [54]. In this review, the authors demonstrated the cohort of patients who more quickly normalized serum lactates had significantly lower mortality. Of note, the included studies that monitored lactate most frequently were still exposed to one- to two-hour monitoring gaps.

On a practical level, given that the half-life of lactate is 15 to 30 min in healthy subjects [59], and that the first and second lactate measurements are often separated by more than four hours, large variations in lactate levels may be missed in routine testing intervals. This implies that each lactate measurement is simply a “snapshot in time”. This blind spot can be addressed with the ability to monitor lactate on a near-continuous basis.

Early warning scores

Acute deterioration of health status is common in hospitalized patients and is often preceded by changes in their vital signs. Currently, gaps exist in the recognition of early deterioration once patients leave the intensive care unit or any other setting where they are being evaluated regularly.

There have been attempts to create early warning scores (EWS) to alert providers to deteriorating patients. These EWS use structured data, including patient demographics, vital signs, and nursing assessments to stratify patients by risk of deterioration [60]. Although verified in a number of different settings and their ability to detect patients in extremis, EWSs lack the discriminatory power to predict deterioration in advance. Examples of EWS include the National Early Warning Score 2 (NEWS2), which uses seven physiological variables; the Epic Deterioration Index, which is a proprietary prediction model; and the MCURES index developed at the University of Michigan using eight variables [61-63]. Increasingly, commercially available solutions are making use of AI technology. Scores differ widely in their accuracy and there have been calls for greater transparency and oversight [64].

The comparative performance of EWS is confounded by the heterogeneity in the datasets and methods used to develop and validate each score. Despite their widespread use, the evidence base for early warning scores remains surprisingly thin. Many scores have serious methodological flaws or have not been externally validated, and relatively few scores are shared openly [65]. Scores are prone to error due to miscalculation or incomplete recordings in part due to time lag in clinical documentation by bedside teams into the EMR, which is a key limiting step for the algorithms.

It is accepted that lactate can improve the prediction of patient deterioration and/or need for ICU admission [3-10]. What is currently missing is combining early warning scores and lactate. The reasons for this omission lie with the current difficulties in obtaining lactate outside of ‘high intensity’ areas of hospitals, such as the ICU or ED. Getting a lactate measurement on the normal floor often involves numerous steps: ordering the test, finding a phlebotomy-competent member of staff, and sending the sample to the laboratory until the result is finally conveyed to a medical decision maker. This process can take several hours, time which deteriorating patients do not have [66].

Combining a standard EWS and lactate is an appealing concept as it brings together a tool for recognizing short-term clinical deterioration with a universally accepted marker for impaired tissue perfusion. There have been attempts to include lactate in NEWS2, creating a NEWS-2L. Studies performed in ED patients and in inpatients on the normal floor showed that such a combined score has better predictive value than the EWS or lactate alone [67-70]. To date, the fundamental problem of having to obtain the lactate level via a slow and sometimes cumbersome chain of events remains the main stumbling block for this combined score.

Future directions

**Figure 2:**
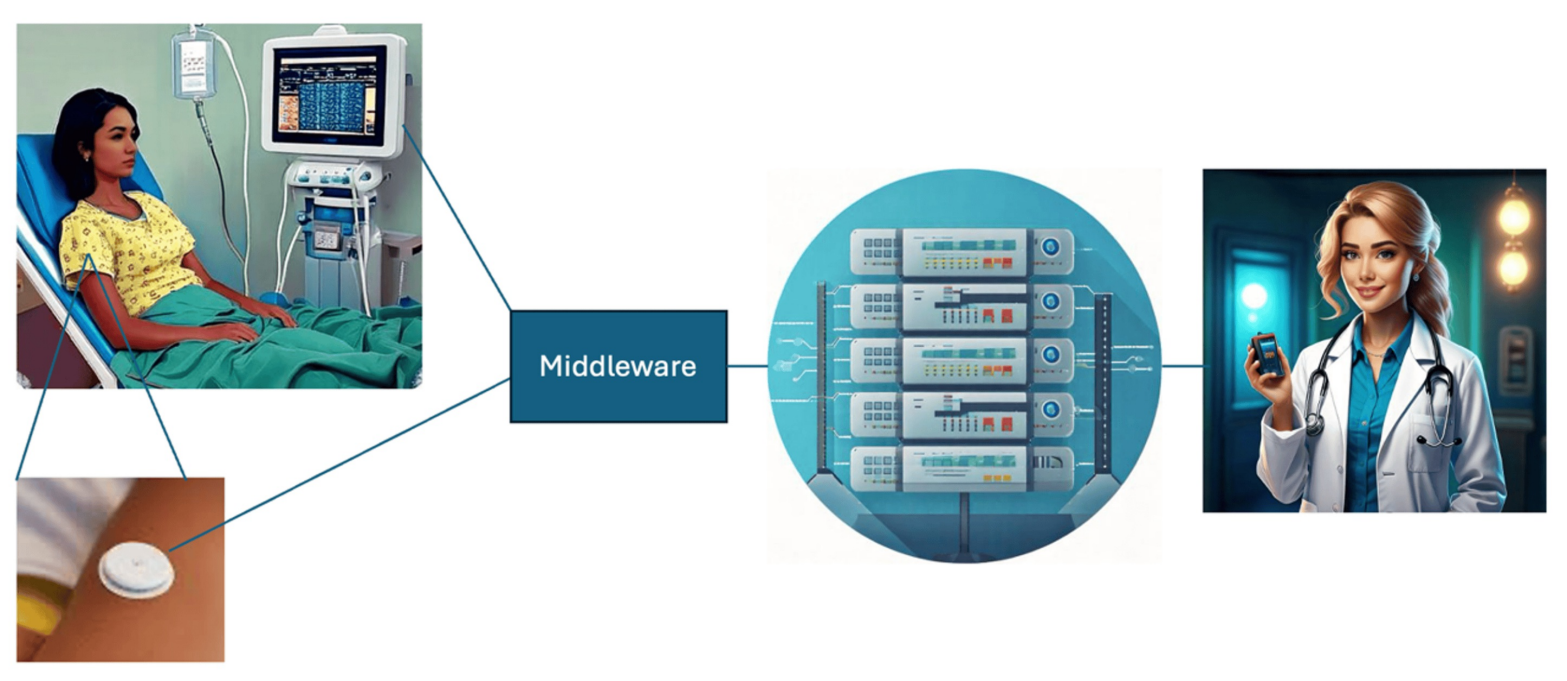
Bedside monitor and lactate sensor continually feed information to hospital middleware, which integrates information received before sending it to the server housing the early warning software package. The software will alert medical team if a patient reaches a predefined trigger. This figure is an original work of the authors. Included images generated by Adobe Firefly Model 3 (Adobe Inc., San Jose, USA), November 1, 2024.

The feasibility and ease of obtaining a combined EWS/lactate score are likely to change once wearable lactate sensors can be introduced into clinical practice. At-risk patients would have the added benefit of lactate surveillance throughout the care period ranging from admission to intensive care, then continued to the hospital ward and discharge. Some hospitals are already equipped with commercially available products that automatically compute parameters grabbed from patient monitors to an EWS and can send out alerts to medical teams when a patient reaches the threshold for a trigger. Integrating the readings from a continually analyzing and emitting wearable lactate sensor via the hospital middleware is technically feasible and easy to achieve. Incorporating lactate into an EWS and setting the points for triggers can either be done manually or will in the future possibly be done with the help of machine learning. Figure 2 shows a schematic drawing of the architecture such a system will require in principle.

A major beneficiary is likely to be the marginal patients on the normal floor. The various constraints mentioned before - staffing availability, delay in entering observations into EMR, and difficulties in obtaining lactate measurements - impact the usefulness of EWS. An automated solution, which does not rely on human input, is more likely to provide a timely and accurate reflection of a patient’s physiological state. 

Table 2 provides a fictitious patient episode that most acute care clinicians from a variety of settings will be more than familiar with. Although the patient showed signs of not being well at 11:06, the then-normal lactate is likely to have instilled a false sense of security in the clinical team. Their condition seems to have improved somewhat over the day before a further deterioration after 20:00. The lactate obtained at this point revealed a marked increase from the one obtained earlier in the day. It can be argued that had there not been a gap of 10 hours between the two measurements, the lactate trend throughout the day would have alerted the medical team to the patient’s progressive but occult deterioration. Earlier intervention could have avoided an admission or re-admission to ICU.

**Table 2:** An illustrative case demonstrating the impact of monitoring gaps that are common when assessing serum lactate levels.

Time	National Early Warning Score (NEWS) 2	Heart rate	Blood pressure	Respiratory rate	Oxygen saturation	Fraction of inspired oxygen	Temperature	Lactate
05:42	5	80	121/84	17	99%	28%	37.1	
06:37	5	78	138/95	20	99%	28%	36.6	
11:06	7	75	97/69	17	99%	24%	36.7	2
12:03	4	74	106/74	17	99%	21%	36.6	
15:21	3	69	118/98	18	99%	21%	36.0	
19:08	3	61	82/43	16	96%	24%	36.1	
19:56	3	59	102/36	20	96%	24%	36.1	
20:42	7	57	99/51	27	96%	24%	36.4	
20:59	7	61	98/38	26	99%	28%	36.2	7.6
21:12	11	80	95/38	26	86%	28%	36.2	
21:51	9	95	78/45	27	99%	100%	35.9	

## Conclusions

The introduction of wearable lactate and glucose monitors into the inpatient setting has the potential to transform hospital medicine by providing continuous, real-time data on patients' metabolic status. Unlike traditional intermittent monitoring, these devices enable clinicians to detect early metabolic shifts associated with conditions like shock and sepsis, allowing for timely and targeted interventions that may prevent clinical deterioration. Continuous lactate monitoring, similar to glucose, would close critical gaps in patient surveillance, offering a new layer of insight into patient physiology that can inform more precise and proactive treatment strategies. Furthermore, incorporating lactate and glucose metrics into early warning systems could greatly enhance their predictive power, shifting these tools from reactive indicators to proactive alert mechanisms.

Realizing this potential will require optimizing wearable sensor technology for clinical settings, ensuring accuracy, and integrating these devices with electronic health records for seamless data access. By prioritizing these efforts, we can make real-time metabolic monitoring as routine and accessible as standard vital signs, reducing the need for invasive testing and minimizing delays in intervention. The path forward demands collaboration across clinical, technological, and regulatory fields, but the benefits are clear: wearable lactate and glucose monitoring can improve patient outcomes, reduce monitoring burdens, and pave the way for a new standard in continuous, patient-centered care. This vision is within reach and represents an essential step toward closing the current gaps in inpatient monitoring.
